# Cost analysis of large-scale implementation of the ‘Helping Babies Breathe’ newborn resuscitation-training program in Tanzania

**DOI:** 10.1186/s12913-016-1924-2

**Published:** 2016-12-01

**Authors:** Sumona Chaudhury, Lauren Arlington, Shelby Brenan, Allan Kaijunga Kairuki, Amunga Robson Meda, Kahabi G. Isangula, Victor Mponzi, Dunstan Bishanga, Erica Thomas, Georgina Msemo, Mary Azayo, Alice Molinier, Brett D. Nelson

**Affiliations:** 1 Departments of Epidemiology and Global Health, Harvard T.H. Chan School of Public Health, Boston, MA 02115 USA; 2 Division of Global Health, MassGeneral Hospital for Children, Boston, MA 02114 USA; 3 Jhpiego, Dar es Salaam, Tanzania; 4 Ministry of Health and Social Welfare, Dar es Salaam, Tanzania; 5 Children’s Investment Fund Foundation, London, W1S 2FT UK; 6 Departments of Pediatrics and Emergency Medicine, Massachusetts General Hospital, Boston, MA 02114 USA; 7 Harvard Medical School, Boston, MA 02115 USA

**Keywords:** Activity-based costing, Cost-analysis, Helping Babies Breathe, Newborn resuscitation, Resuscitation-training, Low-income countries, Tanzania, Low-resource setting, Resource-poor setting

## Abstract

**Background:**

Helping Babies Breathe (HBB) has become the gold standard globally for training birth-attendants in neonatal resuscitation in low-resource settings in efforts to reduce early newborn asphyxia and mortality. The purpose of this study was to do a first-ever activity-based cost-analysis of at-scale HBB program implementation and initial follow-up in a large region of Tanzania and evaluate costs of national scale-up as one component of a multi-method external evaluation of the implementation of HBB at scale in Tanzania.

**Methods:**

We used activity-based costing to examine budget expense data during the two-month implementation and follow-up of HBB in one of the target regions. Activity-cost centers included administrative, initial training (including resuscitation equipment), and follow-up training expenses. Sensitivity analysis was utilized to project cost scenarios incurred to achieve countrywide expansion of the program across all mainland regions of Tanzania and to model costs of program maintenance over one and five years following initiation.

**Results:**

Total costs for the Mbeya Region were $202,240, with the highest proportion due to initial training and equipment (45.2%), followed by central program administration (37.2%), and follow-up visits (17.6%). Within Mbeya, 49 training sessions were undertaken, involving the training of 1,341 health providers from 336 health facilities in eight districts. To similarly expand the HBB program across the 25 regions of mainland Tanzania, the total economic cost is projected to be around $4,000,000 (around $600 per facility). Following sensitivity analyses, the estimated total for all Tanzania initial rollout lies between $2,934,793 to $4,309,595. In order to maintain the program nationally under the current model, it is estimated it would cost $2,019,115 for a further one year and $5,640,794 for a further five years of ongoing program support.

**Conclusion:**

HBB implementation is a relatively low-cost intervention with potential for high impact on perinatal mortality in resource-poor settings. It is shown here that nationwide expansion of this program across the range of health provision levels and regions of Tanzania would be feasible. This study provides policymakers and investors with the relevant cost-estimation for national rollout of this potentially neonatal life-saving intervention.

## Background

An estimated 2.9 million neonatal lives (from birth through day 28) are still being lost each year globally, with the persisting highest risks of death occurring in African countries and within the first 24 h of life [1]. Addressing global standards of care for neonates to reduce these deaths is of increasing importance in accelerating progress toward the fulfillment of global child mortality reduction targets [2]. Neonatal deaths are due in part to a lack of trained birth attendants with basic requisite skills for newborn resuscitation [2]. ‘Helping Babies Breathe’ (HBB) is an evidence-based curriculum devised to meet the training needs of large groups of birth attendants to become skilled in the essentials of neonatal resuscitation, with a focus on achieving adequate ventilation of apneic newborns within the first minute of life – the so-called “Golden Minute” [3].

The American Academy of Pediatrics developed HBB, in partnership with USAID, Save the Children, and UNICEF, amongst others [4]. As HBB becomes the gold standard of care for minimum newborn resuscitation training of all birth attendants globally, both government and non-governmental entities are increasingly focusing on costs and impacts of implementation. Although some studies have investigated the cost-effectiveness of Essential Newborn Care (ENC) and integrated newborn care packages incorporating resuscitation to reduce newborn mortality, very few attempts have been made to date to quantify the costs and effects of newborn resuscitation at scale in resource-poor settings where the potential cost-effectiveness may be invaluable [5–13]. Although ENC expansion alone in resource-poor settings has been investigated, with Manayasan et al. reporting a 41% reduction in neonatal mortality (RR 0.59 95% CI 0.48–0.77), further investigation of asphyxia-related deaths and those potentially preventable through neonatal resuscitation capacity-building warrant further investigation [9].

Although large-scale formal cost-effectiveness analyses have been planned to address expansion of such HBB programs in resource-poor settings [13], our study is the first to fully describe the costs of implementing the HBB program at a regional and national scale. Prior to this study, cost analysis of HBB introduction had been limited to a missionary hospital in Tanzania, in which HBB was found to be a highly cost-effective intervention [6]. By accounting for costs at a regional and national scale in Tanzania, we attempt to account for the diverse scope of service provision and potential for achieving economies of scale for governments considering expansion in comparable resource-limited settings as called for by Msemo et al. [7].

In September 2009, the Tanzanian Ministry of Health and Social Welfare (MOHSW) launched a national HBB training program. Since 2012, through the funding support of the Children’s Investment Fund Foundation and implementation partner Jhpiego, HBB has been implemented at scale in 16 regions throughout Tanzania in a phased region-by-region rollout among a targeted 14,000 facility-based providers. Initial one-day HBB training sessions were held centrally within several districts in each target region with providers from all levels of the health care system. Four to six weeks post-training, follow-up visits were conducted at all of the facilities for program monitoring, equipment assessment, and follow-up appraisal of the trainees’ skills through objective structured clinical examinations (OSCEs) [14]. Additional subsequent follow-up and supportive supervision visits were also provided longitudinally. The Tanzania MOHSW has plans in progress to integrate HBB expansion with ENC, to achieve economies of scale and demonstrate global standards of newborn care.

The purpose of this study was to conduct a cost analysis as one component of a multi-method external evaluation of the implementation of an HBB program at scale in Tanzania. This cost analysis aimed to determine the full costs of initial program implementation in one generally representative region of Tanzania. Any differences in this region with other regions of Tanzania were accounted for in additional scale-up and sensitivity analyses to model what the projected costs would be for program implementation throughout national mainland Tanzania. Further sensitivity analyses are also presented for appraisal of potential maintenance costs over a one- and five-year period, according to the costs of conducting repeated refresher trainings using the current model. As HBB is further expanded across Tanzania and across the globe, these cost data will help to inform stakeholders on the human and financial resources needed to accelerate reductions in neonatal mortality and establish global standards of newborn care [5–12, 15].

## Methods

### Aim

The primary objective of this study was to do a first-ever activity-based cost-analysis of at-scale HBB program implementation and initial follow-up in a large region of Tanzania and evaluate costs of national scale-up as one component of a multi-method external evaluation of the implementation of HBB at scale in Tanzania.

### Study design

The activity-based cost-analysis study utilized real-time cost data collection during a two-month period of program administration in a cross-sectional design in a selected region of Tanzania. Sensitivity analyses were utilized to estimate national costs of scale-up.

### Study setting

The study was purposively conducted in Mbeya Region, which is located in the southwest mainland of Tanzania, is largely rural, and is surrounded by highlands. The regional capital is Mbeya City. Mbeya Region is one of Tanzania’s 25 mainland regions, covering an area of 62,420 km^2^ with a population of 2,707,410 according to 2012 census findings [16]. Within the Mbeya Region, there are a total of 407 health facilities (386 of which are operational), the majority being dispensaries, followed by health centers, and hospitals [17]. The Mbeya Region was purposely selected for regional-level program cost analysis as is thought to be highly representative of the overall variation of urban and rural providers within the mainland Tanzanian healthcare provider landscape. Scale-up analysis is conducted to extrapolate findings to the other regions where HBB is being implemented. Scale-up and sensitivity analyses consider the effects of variation in region characteristics, including geographical area and population density to capture potential cost differences between regions upon national scale up.

### Data collection and cost analysis

The cost data related to training implementation and facility-level follow-up were collected between February and March 2014. Cost-analysis of the program used a micro-costing, bottom-up approach combining activity-based costing (ABC), using real-time budget expense data [18–21]. Activity-based costing is a preferred method in the context of program expansion [21, 22]. Expenditure data were collected from the central, Dar es Salaam-based, Jhpiego program office to determine expenses attributable to the HBB program in Mbeya. Cost data for this study were collected using a series of questionnaires. Staff at the implementing organization headquarters filled out standardized structured forms to obtain real-time cost data on office costs, personnel costs, initial training sessions, refresher trainings, and monitoring visits. Itemization of all individual input activities performed as part of the HBB program (e.g., the initial training sessions, equipment distribution, monitoring visits) was completed using these standardized data collection instruments.

Three categories of costs were considered in this study: (1) program-specific costs, (2) personnel costs, and (3) capital costs. The activity cost centers within these cost categories were itemized as: (1) initial training session and equipment, (2) facility-based follow-up visits 4–6 weeks post-training, and (3) central administration of the program (Tables 1 and 2). Sensitivity analyses account for geographical and economic sources of variation in cost and for costs of maintenance of the program according to repetition of the follow-up visits that would be required over a one-year period to sustain the program and according to the refresher trainings and project continued administration and equipment costs over a five-year period (Tables 3 and 4).

**Table 1:** Cost-activities of national HBB training program implementation and follow-up

Personnel and capital costs	Comments
Cost-activity center A: Central administration	
Leadership Implementation Monitoring Administration Office support	International hires, in-country leadership Inclusive of audit, finance, communications, human resources, operations, procurement, program staff, and transport costs Office space, supplies
Program-specific costs	
Cost-activity center B: Initial training	
Training of birth attendants Distribution of HBB equipment	Ministry of Health and implementation partner costs Trainer and trainee per diems Laerdal NeoNatalie mannequin, and multiple sets of HBB newborn resuscitation equipment (e.g., reusable bag-mask device, reusable suction device, etc.) [26] Training materials (e.g., HBB learner’s manuals, HBB wall poster, HBB flipchart, etc.) Printed material Administration (inclusive of venue costs, associated accommodation, transportation)
Ensuring competence (OSCEs)	
Cost-activity center C: Follow-up visits	
Sustaining training Ensuring skill retention (OSCEs) Verifying presence of HBB equipment	Ministry of Health and implementation partner costs Provider and trainer per diems Printed material Administration (inclusive of associated accommodation, transportation)

**Table 2:** Mbeya Region HBB training program activity-based costs

Activity cost centers	Cost in USD	(Percent)
Cost-center A: Central administration		
Personnel		
Leadership		
International hires	446	(0.2)
In-country leadership	2,201	(1.1)
Implementation staff	16,174	(8.0)
Monitoring team	2,281	(1.1)
Administrative staff		
Audit and finance	14,961	(7.4)
Communications	3,157	(1.6)
Human resources	5,409	(2.7)
Operations department	7,156	(3.5)
Procurement department	3,693	(1.8)
Program staff	2,358	(1.2)
Transport department	2,115	(1.0)
Benefits	13,408	(6.6)
Office space and supplies		
Office space rent	747	(0.4)
Utilities	150	(0.1)
Other contractual costs^a^	598	(0.3)
Office supplies^b^	302	(0.1)
Total	75,156	(37.2)
Cost-center B: Initial HBB training		
Personnel		
Per diem for trainers	9,694	(4.8)
Per diem for trainees	32,066	(15.9)
Per diem for implementing partner	1,725	(0.9)
Per diem for ministry of health staff	203	(0.1)
Equipment [26]		
Mannequins (70 USD each)	24,104	(11.9)
Bag-mask devices (15 USD each)	10,407	(5.1)
Penguin suckers (3 USD each)	2,848	(1.4)
Learner workbooks	13	(0.0)
Training forms (registration, OSCE)	505	(0.2)
Other (communication, stationary)	44	(0.0)
Venue	1,569	(0.8)
Food	5,648	(2.8)
Transportation	1,134	(0.6)
Housing	1,465	(0.7)
Total	91,425	(45.2)
Cost-center C: Follow-up training		
Personnel		
Per diem for trainers	13,857	(6.9)
Per diem for providers	369	(0.2)
Per diem for implementing partner	2,744	(1.4)
Per diem for ministry of health staff	450	(0.2)
Supplies (photocopying)	253	(0.1)
Transportation	14,111	(7.0)
Housing	3,875	(1.9)
Total	35,659	(17.6)
Total Costs for Mbeya Region	202,240	(100.0)

^a^Other contractual costs include delivery services, waste removal, contract cleaning, etc

^b^Office supplies include computer software, printing and photocopying, furniture, etc

**Table 3:** Sensitivity analysis: variation in cost per facility and for all Tanzania rollout given variance in selected cost-influential variables

	Program-specific: distance	Program-specific: equipment	Central administration	Per facility	All Tanzania
Mbeya Region	$88,908	$38,174	$75,156	$602	
National estimates^a^	1,647,444	707,355	1,392,623	602	3,747,422
Initial training duration	-	-	-	-	
+ 1 day	-	-	-	758	4,717,826
+ 2 days	-	-	-	914	5,688,230
Economic variation:					
-5%	1,565,072	671,987	1,322,992	572	3,560,051
-3%	1,598,021	686,134	1,350,844	584	3,634,999
+ 3%	1,696,867	728,576	1,434,402	620	3,859,845
+ 5%	1,729,816	742,723	1,462,254	632	3,934,793
Population coverage:					
65%	1,296,310	556,590	1,095,801	602	2,948,702
75%	1,495,824	642,255	1,264,455	602	3,402,534
90%	1,794,830	770,637	1,517,212	602	4,082,680
95%	1,894,587	813,470	1,601,539	602	4,309,595
Distance from central administration:					
-10%	1,482,700	707,355	1,392,623	575	3,582,678
-5%	1,565,072	707,355	1,392,623	589	3,665,050
+ 5%	1,729,816	707,355	1,392,623	615	3,829,794
+ 10%	1,812,188	707,355	1,392,623	628	3,912,166
Equipment costs:					
-20%	1,647,444	565,884	1,392,623	579	3,605,951
-10%	1,647,444	636,620	1,392,623	591	3,676,687
+ 10%	1,647,444	778,091	1,392,623	613	3,818,158
+ 20%	1,647,444	848,826	1,392,623	625	3,888,893

^a^Based on a 1-day initial training duration

**Table 4:** Maintenance cost-analysis (USD)

		Mbeya Region			All mainland Tanzania^a^
	Costs of refresher training	Central administration^b^	Equipment replacement^c^	Per facility	
	35,659	15,032	7,584	173	1079,821
Maintenance costs					
1 year^d^	71, 318	30.064	7,584	325	2,019,115
5 years^e^	213,954	60,128	30,336	907	5,640,794

^a^Based on 6,226 facilities across all mainland Tanzania at 82.6% coverage of facilities

^b^A 20% proportion of initial central administration costs were included in considerations of program maintenance costs for repeated refresher trainings

^c^A 20% proportion of initial equipment costs were included for potential equipment replacement needed in each subsequent year of the program

^d^Based on repeated refresher trainings at six-month intervals to sustain skills in the first year

^e^Based on annual repeat refresher trainings to sustain skills thereafter

### Personnel costs

Central administration personnel costs were limited to staff of the implementing partner, Jhpiego. These are partially recurring costs and refer to cost-activity center A: central administration. Personnel employed by Jhpiego in Tanzania are involved in many different programs aside from HBB, therefore, their expenses were adjusted to reflect the proportion of personnel time attributable to HBB in Mbeya Region during the two-month implementation period. Personnel included program leadership, implementation staff, monitoring staff, and administrative support. Further roles of central administration staff were itemized in the cost analyses, sub-divided into audit and finance, communications, human resources, operations, procurement, program staff, and transport staff (Tables 1 and 2), with a breakdown of exact costs and their respective proportions for the Mbeya initiation presented (Table 2). Further administration costs were attributable to office space rent, office utilities and supplies, and staff benefits. When considering the maintenance of the program per refresher training conducted, a proportion of these costs would be requisite. It is estimated here that approximately 20% of the initiation central administration costs would be required per refresher training (Table 4).

Program implementation administration costs were again attributable to implementation partner Jhpiego staff with additional assistance of regional and district-level MOHSW leadership. These are partially recurring costs referring to cost-activity centers B and C: initial-training and follow-up training. Roles of program implementation staff are again itemized, sub-divided into per diems for trainers, trainees, implementing partner staff, and ministry of health staff (Tables 1 and 2), with a breakdown of exact cost and their respective proportions for the Mbeya Region initiation presented (Table 2). Cost-activity center C represents a fully recurring cost and provides the basis for the program maintenance-cost analysis, as ongoing program support is based on conducting repeated follow-up trainings in the form of refresher trainings with a proportion of cost-activity centers A and B costs to account for the repeated central administration and resuscitation equipment costs required (Table 4).

### HBB program-specific costs

These included costs incurred exclusively in the implementation of the HBB program in the Mbeya Region. These are partially recurring costs and refer to cost-activity centers B and C: initial training and follow-up training (with cost-activity center C: follow-up training, representing a fully recurring cost as above). These include all expenses attributable to HBB training sessions and facility-based follow-up visits, specifically, costs related to training equipment, rental of a training venue, food, transportation, and accommodations. Program-specific costs were differentiated from personnel and capital-costs, both of which may be shared with Jhpiego programs other than HBB and, therefore, represented a proportion of their central office costs. The significance of program-specific costs to decision-makers lies in that they must be regularly renewed as are accounted for in the maintenance analysis presented below (e.g., in the form of refresher trainings and equipment replacement). Equipment replacement was factored into the analysis of maintenance costs, assuming up to 20% of equipment would require replacing per year following initiation (Table 4).

### Capital costs

Capital costs were itemized within cost-activity center A: central administration. This included vehicles, office infrastructure, computers, office furniture, and other assets required for the functioning of Jhpiego’s central office in Dar es Salaam and proportionally attributable to the two-month implementation of HBB in Mbeya.

### Equipment costs

Equipment costs were itemized within cost-center B: initial training, as in general these costs are not anticipated to be recurring, but a proportion of replacement costs are accounted for in program maintenance cost projections. Resuscitation equipment included Laerdal NeoNatalie mannequins and multiple sets of reusable bag-mask and suction devices, according to the size of the facility. Training materials (e.g., HBB learner’s manuals, HBB wall poster, large HBB flipchart, etc.) were also provided.

### Sensitivity analyses

Finally, sensitivity analyses were conducted to demonstrate how potential variations in variables across settings might impact overall costs (Table 3). Sources of variation in program-specific and administrative costs are anticipated according to differences in regional economic variation, distance between the region and central administration, population coverage, and implementation by local ministry of health or an international non-governmental organization. Equipment costs are anticipated to vary across settings, through economies of scale, and may reduce over time as advances are made in production.

### Maintenance costs

Additional sensitivity analyses were undertaken to estimate those costs that would be incurred for sustaining the training through repeated refresher trainings and the attendant administration and equipment costs over a one- and five-year period (Table 4). A projected 20% of the program initiation costs was estimated as effort needed for central administration to deliver refresher training. All central administration costs were based upon the costs needed of the non-governmental implementation partner to conduct work. All central administration costs and maintenance administration costs are, therefore, proportionally attributable to the duration of activity needed by a central support mechanism. A projected 20% of initial equipment costs is used to estimate the annual cost of replacing equipment in maintaining the program, assuming a loss of function in some of the materials over time.

## Results

### Cost analysis

The total cost for implementation of the HBB training program in the Mbeya Region over a two-month period in 2014 was $202,240. This included total initial training costs of $91,425, total follow-up visit costs of $35,659, and total central administration costs of $75,156. In total, 49 training sessions were undertaken, involving the training of 1,341 health providers from 336 health facilities in Mbeya Region, such that the cost of delivering HBB training at the regional level was $4,128 per training session, costing $151 per trainee and $602 per health facility.

### Coverage of the HBB training program

A total of 336 of 407 (82.6%) health facilities in the Mbeya Region participated in the trainings, with an average of four providers from each facility, ranging from one to nine, depending on the level of facility. During the facility-based follow-up visits, the Jhpiego program assessed a total of 1,001 health providers from 322 of these 336 trained health facilities (95.8%). Ten of the 11 (90.9%) district councils were also visited.

### Cost distribution

Of the overall total program costs for implementing HBB in Mbeya Region, the highest proportion of costs was spent on initial training costs (45.2%), followed by central administration costs (37.2%), and lastly follow-up visit costs (17.6%) for program initiation.

### Scale-up costs

All scale-up estimates were based on the best-available national data for scaling to the 25 Tanzanian mainland regions. The five additional regions of Zanzibar are under the leadership of a separate ministry of health and, as island regions, were considered to have unique costing issues and, therefore, are not included in the analyses. However, the estimates presented here rely on assumptions, which are explored further in the sensitivity analyses and discussed as limitations to this study below.

### Per health facility

According to national data, there are a total of 7,537 health facilities (6,640 of which are operational) within the Tanzanian mainland.^7^ Using per-facility costs calculated in this study, scale-up to 6,226 (82.6% coverage, as with Mbeya Region) of these facilities would cost an estimated $3,747,429.

### Per training session

Forty-nine trainings were required to reach 336 health facilities. Coverage of 82.6% of the total national number of health facilities would require an estimated 908 training sessions, costing a projected $3,747,579.

### Per trainee

Assuming roughly four healthcare providers trained in HBB per health facility, and assuming 82.6% coverage, the cost of scaling-up training to reach 24,904 providers would be $3,755,772.

Calculations of national costs based on per-facility, per-training, and per-trainee costs in Mbeya Region were fairly consistent. The overall range in these various per-item estimates suggests a minimum potential cost of $3,747,429 and a maximum potential cost of $3,755,772 to cover approximately 82.6% of mainland Tanzania. The effects of possible sources of variation are explored in brief in the following sensitivity analyses.

### Sensitivity analyses

Extension in duration of initial training would incur the greatest potential impact on overall national roll out costs. Within regional cost of initial training (cost-center B; Tables 1 and 2), 57.3% of the costs were calculated to be recurring (personnel, venue, food and housing; Table 2). Hence, costs for extended duration of initial training would cost to the national rollout an estimated addition of $970,470 per additional day of initial training (Table 3). Variation in program costs and personnel costs between regions – for example, as a result of attainable population coverage and distance between the region and the national capital – may additionally incur significant impacts on national scale-up (Table 3). The majority of overall personnel costs, including central administration and program-specific personnel, were attributable to program implementation staff, with a further 28.9% to administrative support, 2.0% to leadership, 1.7% to monitoring and evaluation staff, and the remaining 10.0% to fringe benefits.

Economic variation, including alterations in interest rates and the value of the currency (estimated within the range of −5% to +5% of current), may incur changes to all elements of the program costs, such that overall costs of national rollout may vary from between $3,560,051 and $3,934,793. The extent to which expansion of HBB training is achieved, in terms of the number of regional facilities supplied with trained birth attendants and the consequent coverage of the population with HBB services, influences all elements of program costs. Variation of this coverage from 65% to 95% of the total population may lead to a variation in the total cost of national rollout of the range of $2,948,702 to $4,309,595. Changes in the distance between a region and central administration, as well as the population density of the region, may impact costs of transportation, program-specific personnel as they may need to travel for more days, as well as the costs of the venue, food, and housing. Variation in regional distance from central administration may incur variation in all Tanzania program rollout costs in the range of $3,582,678 to $3,912,166. Variation in equipment costs ranging from −20% to +20% – depending on, for example, economy of scale, would be expected to incur impacts on program supply costs, such that the total Tanzania program cost may vary from between $3,605,951 and $3,888,893.

### Maintenance costs

Analyses are presented for costs of repeated refresher trainings over a one-year and five-year duration following HBB program initiation. Costs of repeated refresher training are assumed to be consistent with follow-up training costs during the initiation. Over this period, it is anticipated that the in-country Ministry of Health would assume responsibility for the program to achieve economies of scale resulting in a reduced regular investment of 20% of the initial central administration costs for each refresher-training. Twenty percent of initial equipment costs are anticipated for replacements for each year following initiation. It is, thus, estimated that it would cost around $173 per facility for a single refresher training session ($1,079,821 for all Tanzania) and hence $2,019,115 to sustain the program nationally for one year based on twice yearly refresher visits in the first year, and $5,640,794 to sustain the program nationally for five years based on annual refresher trainings thereafter.

## Discussion

HBB is considered one of the leading interventions for improving health outcomes in low- and middle-income countries [23]. As HBB is further expanded across the globe, these at-scale cost data will be an essential tool providing stakeholders with critical information on the human and financial resources needed to deliver reductions in perinatal mortality. Our calculations project a cost to implementing an HBB training program at $4,128 at the regional level, serving around seven health facilities at a cost approximating $602 per health facility. We estimate each re-training to cost $1,211 for seven health facilities ($173 per facility). Voissus et al. found in a single hospital site in Tanzania that initial training cost $2,084 and re-training cost $1,515 [6]. Our cost data capture cost-influential factors involved in rolling out an HBB program at a regional level, we expect these data to be a reasonable estimate of regional costs of scale-up in regions comparable to Mbeya Region. Our national estimate of $4,000,000 is further examined in sensitivity analyses to consider the effects of variations in cost-influential variables across the differing regions of Tanzania, giving bounds to this estimate of $2,934,793 to $4,309,595. Our cost estimates are based on a one-day initial training period. Further estimation of the impact of extended duration to the initial training is considered to have the greatest overall impact on potential costs, at an excess of just under $1,000,000 per day. Additional cost, therefore, is a consideration in decisions regarding the optimal duration of the initial training period in resource-limited settings.

Resuscitation training of birth attendants within integrated newborn care packages along with other evidence-based measures to save lives have resulted in significant reductions in neonatal mortality [5–12]. Carlo et al. did not find a reduction in mortality following introduction of newborn resuscitation training, however, the resuscitation training was conducted after ENC training that included elements of resuscitation training, diluting any potential effects [12]. Bang et al. reported significant reductions in neonatal case-fatality due to severe asphyxia (by 45%, from 39 to 20% (*p* < 0.07)) and asphyxia-related neonatal mortality (by 65%, from 11 to 4% (*p* < 0.02)) in a study of community health workers in India trained in both newborn care and resuscitation skills [24]. The FIRST BREATH trial estimated reductions to be as high as a 30–40% [12]. Similarly, Sabin et al. estimated a 45% reduction from a combined program (RR 0.55 95% CI 0.33–0.90) [5]. Countries implementing HBB and newborn care programs are anticipated to benefit from the systems approach to training, such that expansion of HBB may be conveniently undertaken in conjunction with ENC and other interventions for full potential neonatal mortality impacts and cost-savings to be realized [25].

This study in Tanzania represents a first-ever cost-analysis of implementing the HBB training program at scale. Regional costs were calculated to project cost estimates for national rollout of the program. Data were captured in a real-time effort to expand HBB across Mbeya Region. This is the first report of a regional cost-analysis in a low-income country setting. Activity-based costing methodology, used in combination with the bottom-up costing and ingredients approach gives an accurate measure of the costs involved at the regional level, as well as a valid basis from which to estimate national initiation and maintenance costs for the HBB program. Costs for the HBB program were divided into three activity-cost centers – initial training and equipment, facility-based follow-up visits, and central administration – and into three cost categories – program-specific costs, personnel costs, and capital costs. Of these, initial training costs were found to be the highest, and capital costs were the lowest. Within the training costs, personnel costs were the highest, followed by the cost of HBB training-related equipment.

### Limitations

Costs within this study are estimated under the assumptions of a model of implementation largely administrated by non-governmental organizations and, hence, reflect higher organizational personnel and administration costs. Although there is little available data on the comparative costs of governmental versus non-governmental expenditure in such programs, national ministries of health would be in a position to achieve significant cost savings than are achievable working with a non-governmental implementation partner, to reduce the costs of program expansion as compared to what is presented here. Additional economies of scale could be achieved on larger bulk-purchase of equipment. In the current analysis, the average cost of equipment was $350 per facility in order to provide facilities with multiple sets of training materials, including a Laerdal NeoNatalie mannequin, HBB learner’s manuals, wall posters, and multiple sets of HBB newborn resuscitation equipment (e.g. reusable bag-mask device, reusable suction device, etc.) according to the size of the regional facility [26]. Furthermore, integration of HBB within a package of ENC and other essential neonatal services would likely further reduce overall central administration costs, whilst increasing health gains and consolidating a systems based approach to neonatal healthcare [25]. Empirical data on the extent of cost-savings achievable through government administrated, integrated delivery of care at high volume, such that economies of scale could be fully realized, within resource-limited settings are warranted. Such sources of potential cost-savings on overall national HBB program rollout and sustainability costs were not immediately estimable within the limits of the current study.

For this analysis it was necessary to extrapolate real time cost data from Mbeya Region in order to make estimates for scale-up to all of the other mainland regions. While sensitivity analyses served to apply this regional information to projected national implementation, these projections necessarily rely on assumptions that the cost of program implementation in Mbeya Region is representative of other regions within Tanzania and at different times. Efforts to account for major sources of potential variation in costs regionally and over time are presented in the sensitivity analyses. The findings of the sensitivity analysis, suggest that the national estimate for program initiation we project of around $4,000,000 is potentially robust to several sources of cross-regional differences in cost-influential variables. We consider the influence of economic variations such as interest rate and currency valuation changes, population coverage achievable across regions according to geographical area and population density, distance of the region from the site of central administration, as well as potential variations in the costs of equipment. However, our choice of cost-influential variables warrant further investigation, as there is uncertainty regarding the true effects of variation across regions upon national expansion of the program.

Regional expansion presented here achieved 82.6% coverage of facilities and 74.6% coverage of providers. This represents a highly satisfactory proportion of coverage for an at-scale intervention in a real-world low-income country setting, considering the variation in health-services anticipated across regions and our choice to represent all facilities (total versus operational). For instance, in typical lower-level facilities such as dispensaries, there may be only 1–2 skilled birth attendants on staff such that releasing staff to attend a centralized training at the district hospital can be difficult in conjunction with continued delivery of clinical care. Additionally, HBB-trained providers can be rotated to new departments, and new staff are hired. These result in a dilution of the training coverage.

This study did not attempt to account for program planning and start-up costs, such as the training of the MOHSW master trainers who conducted the regional training sessions. MOHSW leadership contributions were also not included. Furthermore, scale-up analysis is based upon the number of national health facilities information from the Tanzanian MOHSW. We chose to use total facilities, as opposed to operational facilities, to provide a conservative estimate for national rollout.

In the absence of a formal cost-effectiveness analysis at scale, and randomized control trial data to demonstrate the intervention effect size in this context, it is challenging to reliably compare the costs and impacts to similar neonatal care programs. Additionally, there are no reliable baseline measurements of mortality concurrent with these cost measurements. Data are needed on mortality indicators associated with HBB implementation at scale, such as number of lives saved or number of resuscitations conducted, without which it is not possible to estimate the cost per neonatal death averted or disability-adjusted life year gained.

### Sustainability

Maintenance costs arising from sustaining the HBB program following initial rollout have been previously estimated to be significantly lower than initial rollout costs – approximately one-third the costs of implementation per year [6]. The consideration of maintenance costs here is essential to ensure further uptake and retention of skills over time, through activities such as refresher trainings. The activity-based costing methodology allows for efficent calculations of valid maintenance costs, however, some assumptions are necessary for considering the proportion of administration and equipment replacement costing. These, unfortunately, are untestable assumptions but are in keeping with expectations established from prior studies [5–12].

Our cost data demonstrate the financial feasibility of HBB regional and national expansion and maintenance over one and five years in low-income country settings. For contextualization of the magnitude of the costs, we consider that the latest national health budget in Tanzania was approximately $800 million [27]. Therefore, countrywide implantation of a $4 million HBB program would account for roughly one half percent of the country’s annual health system budget. Given an approximate projected $2 million cost for one-year program maintenance and $6 million cost for five-year program maintenance, the countrywide expansion and sustenance of such newborn standard of care practices is highly cost-feasible.

Further efforts to quantify the costs and impacts of integrated packages of neonatal care involving essential care are called for. Although estimation of these is beyond the scope of this work, this cost analysis does provide a useful framework for policymakers to estimate the potential costs invovled in expanding programs of newborn care, based on similar models of training as HBB is designed to be easily ammended with additional training elements and is intended to provide a platform for training that enhances a systems-approach to delivery of care [3–13, 24, 25].

## Conclusion

National rollout of the HBB program in Tanzania is financially feasible. Stakeholders can use the current study as a guide for costing out the expansion of this potentially life-saving neonatal resuscitation program in other resource-limited settings. Formal cost-effectiveness analyses are warranted to assess potential cost-savings per neonatal death averted.

## Abbreviations

ABC: Activity-based costing
ENC: Essential Newborn Care
HBB: Helping Babies Breathe
MOHSW: Ministry of Health and Social Work
OSCE: Objective structured clinical examination
USAID: United States Agency for International Development
UNICEF: United Nations Children’s Fund
